# Supplementary material for: Estimating the distance to an epidemic threshold
Source: J R Soc Interface. 2018 Jun 27;15(143):20180034. doi: 10.1098/rsif.2018.0034 (PMC6030631; doi:10.1098/rsif.2018.0034)
Supplement: Supplementary Material [file rsif20180034supp1.pdf]

Supplementary material: Estimating the distance to an epidemic threshold

Eamon B. O'Dea Andrew W. Park John M. Drake

June 9, 2018

Interpretation of the distance to the epidemic threshold when $\eta > 0$

When there is a small rate at which individuals can be infected from other populations or an environmental source ($0 < \eta \ll 1$), there is no longer a disease-free equilibrium but the concept of an epidemic threshold is still relevant. One can see from equation (2.6) that Y^* is slightly increased by the addition of η to the force of infection. When R_0 is not too close to 1, Y^* can be well-approximated by making a linear approximation to the square root function starting at the point where its argument is equal to its first term, which yields

$$Y^*/N_0 \approx \max\left(0, \frac{\mu}{\beta}(R_0 - 1) - \frac{\eta}{\beta}\right) + \frac{\eta}{|\beta - \gamma - \mu - \eta(\gamma + \mu)/\mu|} \tag{S1}$$

$$\approx \max\left(0, \frac{\mu}{\beta}(R_0 - 1)\right) + \frac{\eta}{|\beta - \gamma - \mu|}. \tag{S2}$$

Using this approximation, one can see that when $R_0 \ll 1$, Y^*/N_0 scales with η. When $R_0 \gg 1$, Y^*/N_0 is potentially much larger and effectively independent of η. Also, a large epidemic is only possible when $R_0 \gg 1$. Therefore, we can still consider $R_0 = 1$ as an epidemic threshold for small η. To clarify that Y^*/N_0 is not too large when $R_0 \approx 1$ we need a second approximation. In this case, Y^*/N_0 can be bounded by applying the triangle inequality to obtain

$$Y^*/N_0 < \max\left(0, \frac{\mu}{\beta}(R_0 - 1) - \frac{\eta}{\beta}\right) + \sqrt{\mu\eta/[(\gamma + \mu)\beta]}. \tag{S3}$$

This bound on Y^*/N_0 scales with $\sqrt{\eta}$. Accordingly, Y^*/N_0 is intermediate in size to those cases when R_0 is far from 1. Thus, although when $\eta > 0$ the model never passes through an epidemic threshold, as R_0 passes through one Y^*/N_0 behaves similarly to the case where the model parameters do pass through a threshold point (*i.e.*, when $\eta = 0$). Thus it is still of interest to establish how close the parameters are to the point of the epidemic threshold.

Calculation of perturbations that allow for distance estimate

We here describe a specific calculation for finding the values of D with which variable i can provide a distance estimate. The main step is to find the set of D for which the lag-τ autocorrelation of variable i is within ϵ of a reference function having the same rate of decay and frequency of oscillation as the autocorrelation of the informative eigendirection. The reference function may differ from the autocorrelation function along the informative eigendirection in its phase angle. We work with the autocorrelation instead of the autocovariance because the autocorrelation always ranges from one to zero, which makes a single choice of ϵ correspond to a comparable error for variables with much different variances. To obtain the reference function, we make use of the contribution of the autocovariance along the informative eigendirection to the autocovariance of variable i. If follows from $\Sigma_\tau = W\tilde{\Sigma}_\tau W^\intercal$ that

$$\sigma_{ii,\tau} = \sum_{k=1}^{n}\sum_{k'=1}^{n} w_{ik}w_{ik'}\mathrm{e}^{\lambda_k\tau}\tilde{\sigma}_{kk'}, \tag{S4}$$

where n is the dimension of the phase space. If we restrict k and k' to the indices m and m' of the informative eigenvalues we obtain a reference autocovariance

$$r_{ii,\tau} = \sum_{k\in\{m,m'\}} \sum_{k'\in\{m,m'\}} w_{ik} w_{ik'} \mathrm{e}^{\lambda_k \tau} \tilde{\sigma}_{kk'}. \tag{S5}$$

$r_{ii,\tau}$ states what the autocovariance of variable i would be if the contribution of eigendirections that were not informative to the variance of i were zero. The coefficients $w_{ik}w_{ik'}$ in $r_{ii,\tau}$ can change the value and phase angle of $r_{ii,\tau}$ when $\tau = 0$ relative to the autocovariance along the eigendirections, but they preserve the magnitude of the frequency of oscillation and the rate of decay with τ. We can obtain a reference autocorrelation function by dividing by the autocovariance with $\tau = 0$, which we denote r_{ii}. Therefore, we can express the problem as finding the matrices D that satisfy

$$\epsilon \geq |\sigma_{ii,\tau}/\sigma_{ii} - r_{ii,\tau}/r_{ii}|. \tag{S6}$$

Note from equations (S4) and (S5) that if $n = 2$ and the informative eigenvalues are a complex conjugate pair, then the reference function always equals $\sigma_{ii,\tau}/\sigma_{ii}$ and any covariance matrix D will satisfy equation (S6). If $n > 2$ and the informative eigenvalues are a complex conjugate pair, then equation (S6) is quadratic in some of the elements of D and therefore not trivial to solve. However, if the informative eigenvalue is real-valued, then equation (S6) is linear in the elements of D. In this case we can restate our problem as

$$\epsilon \geq |\sigma_{ii,\tau}/\sigma_{ii} - \mathrm{e}^{\lambda_m \tau}|, \tag{S7}$$
$$\epsilon\sigma_{ii} \geq |\sigma_{ii,\tau} - \mathrm{e}^{\lambda_m \tau}\sigma_{ii}|, \tag{S8}$$
$$-\epsilon\sigma_{ii} \leq \sigma_{ii,\tau} - \mathrm{e}^{\lambda_m \tau}\sigma_{ii} \leq \epsilon\sigma_{ii}, \tag{S9}$$
$$\varepsilon\sigma_{ii} = \sigma_{ii,\tau} - \mathrm{e}^{\lambda_m \tau}\sigma_{ii}, \qquad \forall -\epsilon \leq \varepsilon \leq \epsilon. \tag{S10}$$

To make the linear nature of the problem explicit, we introduce the vector $\tilde{\mathbf{v}}$ which collects the lower triangle of $\tilde{D}$ in a column vector. To be more precise, let $\tilde{v}_\ell$ be equal to the element of $\tilde{D}$ corresponding to the ℓth member of the lexicographic ordering of the set of index pairs (i,j) of $\tilde{D}$ such that $i \leq j$. We may then state the problem as

$$\varepsilon \mathbf{a} \cdot \tilde{\mathbf{v}} = \mathbf{b} \cdot \tilde{\mathbf{v}}, \qquad \forall -\epsilon \leq \varepsilon \leq \epsilon, \tag{S11}$$
$$0 = (\mathbf{b} - \varepsilon\mathbf{a}) \cdot \tilde{\mathbf{v}}, \qquad \forall -\epsilon \leq \varepsilon \leq \epsilon, \tag{S12}$$

where we have first used equation (2.14) and $\tilde{d}_{ij} = \tilde{d}_{ji}$ to write $\sigma_{ii,\tau}$ in terms of the elements of $\tilde{\mathbf{v}}$. We have then collected the coefficients of the elements of $\tilde{\mathbf{v}}$ on the left and right sides of equation (S10) in the vectors $\mathbf{a}$ and $\mathbf{b}$. We thus obtain equation (S12), which is the equation for a plane in the basis corresponding to the elements of the lower triangle of $\tilde{D}$. The plane passes through the origin and has a normal vector that is equal to the vector pointing from $\varepsilon\mathbf{a}$ to $\mathbf{b}$. As ε varies from $-\epsilon$ to ϵ, the normal vector rotates and the union of all of the corresponding planes defines the set of all $\tilde{\mathbf{v}}$ that satisfy equation (S10). Now let $\mathbf{v}$ denote a column vector that collects elements in the lower left triangle of D, and let M be a change of basis matrix that satisfies $\mathbf{v} = M\tilde{\mathbf{v}}$. The elements of M are determined by the relationship $D = W\tilde{D}W^{\intercal}$. The region of space satisfying equation (S10) in the basis corresponding to the lower triangle of D is then given by $0 = (M^{-\intercal}(\mathbf{b} - \varepsilon\mathbf{a})) \cdot \mathbf{v}$ for all $-\epsilon \leq \varepsilon \leq \epsilon$. Thus we have a compact expression for the set of all D for which the autocorrelation of i may be consistent with that of the reference function at a particular lag. The set of all such feasible D may be a subset of the D that satisfy equation (S6) because variance-covariance matrices have additional constraints on their elements.

Of course, if the decay rate of the autocorrelation is what will be used to determine the distance to the threshold, it is important for the autocorrelation of i to be close to the reference function at multiple lags. In general, one could find the D that ensures this by calculating the set of suitable D at each of several lags and finding the intersection of those sets. In the case of a two dimensional system, there is a single lag at which any deviation in the autocorrelation of variable i from that of the reference function is maximized. To see this, observe that in this case $\mathbf{b}$ has one component that is 0 and two other components which include the factor $|\mathrm{e}^{\lambda_1\tau} - \mathrm{e}^{\lambda_2\tau}|$. Those factors are the only places that τ enters into the normal vector components.

Thus τ determines the magnitude of $\mathbf{b}$. This magnitude is proportional to the angles between the vector pointing from $\pm\epsilon\mathbf{a}$ to $\mathbf{b}$ and the vector pointing from $\pm\epsilon\mathbf{a}$ to $\mp\epsilon\mathbf{a}$. Those vectors are the normal vectors of the planes that bound the region in which the autocorrelation is within ϵ of the reference function. As the magnitude of $\mathbf{b}$ approaches zero, the vectors approach being negatives of each other and almost all possible D result in an error less than ϵ. This result is consistent with the intuition that for sufficiently large or small lags, all exponentially decaying functions of the lag with the same initial value will be close to each other. As the magnitude of $\mathbf{b}$ grows, the boundary plane normal vectors both rotate toward each other and the region of suitable D contracts into itself. Therefore, to ensure that the autocorrelation of variable i is within ϵ of the reference function for all lags, it suffices to find suitable D when $|\mathbf{b}|$ is maximal, which corresponds to $\tau = (\log|\lambda_2| - \log|\lambda_1|)/(\lambda_1 - \lambda_2)$.

Equations for autocorrelations

We can use the analytic approach described in Methods of the main text to more rigorously support the claim that the distance to the threshold typically may be estimated only from Y when R_0 is much less than 1. When $R_0 \ll 1$, if follows from equations (2.8) and (2.9) that the eigenvalues of F are $\lambda_1 = -\mu + O(\eta)$ and $\lambda_2 = \beta - \gamma - \mu + O(\eta)$. $O(\eta)$ denotes a term with a coefficient of η which we can expect to be small due to the fact that we restrict η to be small. We take $|\lambda_2|$ as the distance to the threshold. For the autocorrelation of X, we obtain

$$\frac{\sigma_{\tau,11}}{\sigma_{11}} = \frac{\sum_{ij} \mathrm{e}^{\lambda_i \tau} \tilde{\sigma}_{ij}}{\sum_{ij} \tilde{\sigma}_{ij}}, \tag{S13}$$

where

$$\begin{aligned} \tilde{\sigma}_{11} &= \frac{(\beta-\gamma)^2 d_{11} + 2(\beta-\gamma)\beta d_{12} + \beta^2 d_{22} + O(\eta)}{2\lambda_1}, \\ \tilde{\sigma}_{12} = \tilde{\sigma}_{21} &= \frac{(\gamma-\beta)\beta d_{12} - \beta^2 d_{22} + O(\eta)}{\lambda_1 + \lambda_2}, \\ \tilde{\sigma}_{22} &= \frac{\beta^2 d_{22} + O(\eta)}{2\lambda_2}. \end{aligned} \tag{S14}$$

For many infectious diseases of humans $\mu \ll \gamma$. Therefore when $R_0 \ll 1$, $|\lambda_1| \ll |\lambda_2|$. Further, from equation (2.9) d_{11} will by far be the largest element of D for X sufficiently greater than Y. These two conditions imply that for typical parameter choices, $\tilde{\sigma}_{11}$ is by far the largest element of $\tilde{\Sigma}$. Then from equation (S13), the autocorrelation of X should be close to $\mathrm{e}^{\lambda_1 \tau}$. For the autocorrelation of Y, by neglecting $O(\eta^2)$ terms we obtain

$$\frac{\sigma_{\tau,22}}{\sigma_{22}} = \frac{(\gamma-\beta)^2 \mathrm{e}^{\lambda_2 \tau} \tilde{\sigma}_{22} + O(\eta)}{(\gamma-\beta)^2 \tilde{\sigma}_{22} + O(\eta)}. \tag{S15}$$

Clearly, for sufficiently small η the autocorrelation of Y will be close to $\mathrm{e}^{\lambda_2 \tau}$. Thus the value of the decay rate of the autocorrelation of Y provides an estimate of the distance to the threshold when $R_0 \ll 1$ and η is sufficiently small.

Effects of population size on distance estimates

Because the linear noise approximation from which our distance estimates are derived becomes poorer as population sizes decreases, we examined with simulation how the accuracy of distance estimates declined with population size. Figure S1 shows that we found the sensitivity of the accuracy to depend on R_0. In small populations, stochastic elimination of the disease occured frequently, which made the autocorrelation function much different from the prediction made for large populations. Consequently, the distance was consistently overestimated for populations less than 10^5 when $R_0 = 16$ (fig. S1). On the other hand, accuracy was similar across all population sizes when $R_0 = 0.1$. It seems that the Gaussian solution based on large population sizes can still provide a good approximation to the autocorrelation when the population size is fairly small. In short, it seems that in some cases our methods may be reliable with data from population sizes as small as 100.

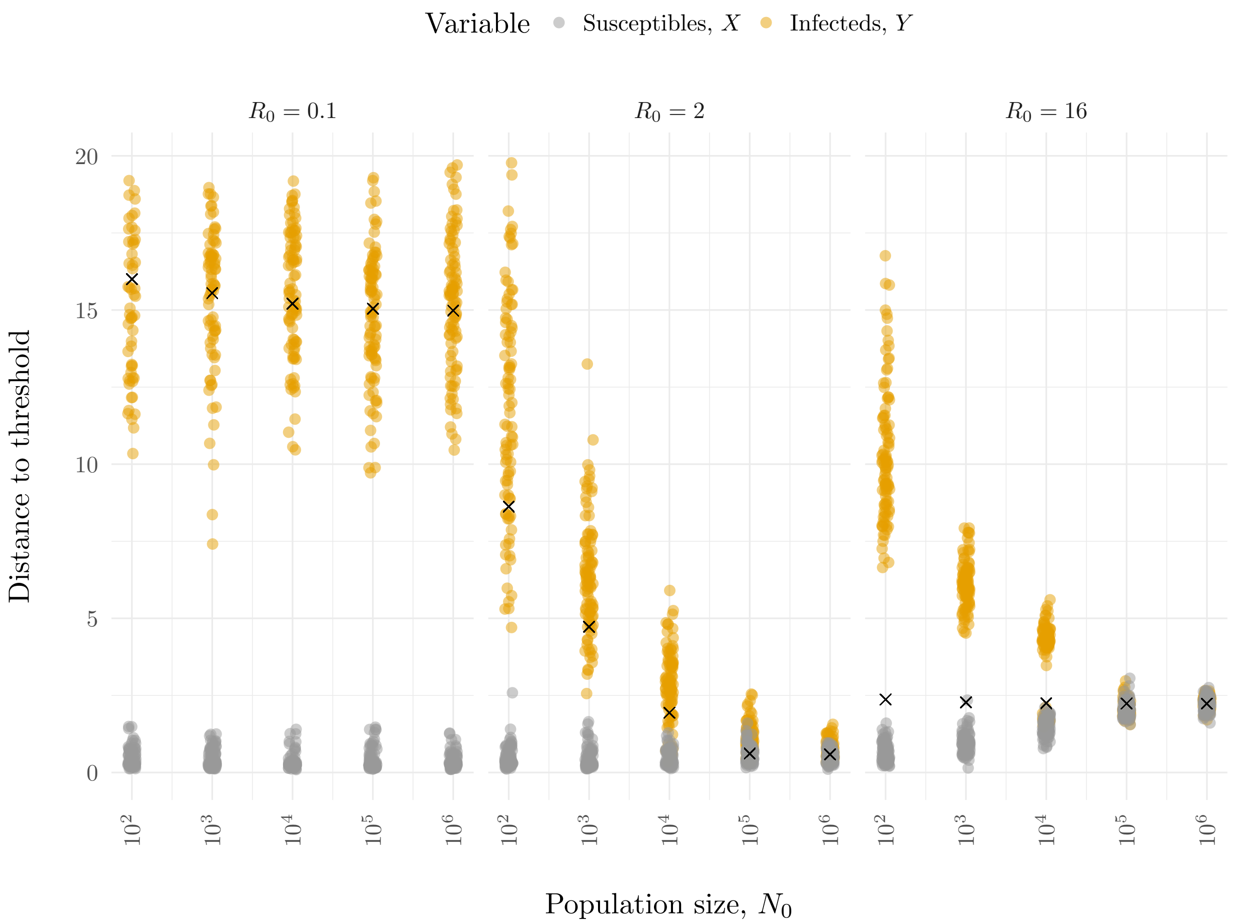

Figure S1: The sensitivity of the accuracy of distance estimates to population size depends on R_0. The true value is marked with an 'x'. One hundred data sets were simulated for each set of parameters and all estimates obtained for each variable are plotted. Parameters besides the population size are in table 1,with β set to $R_0(\gamma + \mu)$.

Effects of environmental noise on distance estimates

Although we found that observation of the number infected could provide accurate distance estimates when the system is subject to intrinsic noise only, figure 2 indicates that deviations from intrinsic noise can change the situation. Thus we next examined the distance estimates with increasing amounts of environmental noise in the death rate. As expected, the $R_0 = 0.1$ panel of figure S2 shows that for sufficiently large amounts environmental noise, the estimates of distance based on Y become inaccurate. However, the amount of noise required to seriously compromise accuracy is large. Figure S3 shows that environmental noise is slow to change the decay of the autocorrelation at short lags, and thus the robustness of the accuracy is a consequence of our distance estimates being sensitive to the rapid decay at short lags. Note that a typical approach when calculating early warning indicators is to look for trends in the autocorrelation at a single lag, and for large enough lags trends in such indicators could mistakenly identify increasing noise in the death rate for an approach to an epidemic threshold. However, we have also noticed that for 20 year time series the increases in the autocorrelation due to environmental noise are typically below their long-term expected value (figure S4), which serves to reduce the effect relative to that expected from figure S3 and also contributes to the robustness of our distance estimates. If the estimated autocorrelations from the 20 year time series looked like those in figure S3, the estimates based on Y would be much more similar to those based on X when the standard deviation in the death rate was 1 and the population size was 10^9.

From the definition of the m terms in equation (2.9), it can be seen that environmental noise in the death rate increases the covariance of perturbations of X and Y. In contrast, environmental noise in the force of infection makes that covariance more negative. To more thoroughly examine the effects of deviations from pure intrinsic noise we also evaluated distance estimates from simulations with environmental noise in the force of infection. Estimates in He *et al.* [1] for the standard deviation of such noise from measles case reports range from 0.038 to 0.096. Estimates from the analysis of polio case reports in Martinez-Bakker *et al.* [2] for the standard deviation of noise in the force of infection range from $1.6 \cdot 10^{-3}$ to 0.68 (personal communication with P. Rohani). Figure S5 plots distance to threshold estimates from simulations having noise levels that bracket these noise parameter estimates. In contrast to the case of noise in the death rate, distance estimates are not affected by high levels of noise when $R_0 = 0.1$, but they are affected when R_0 is equal to 2 or 16. We inspected the time series in these cases and noticed that the variation in the force of infection leads to frequent interruption of the regular cycles that characterize the dynamics in the presence of intrinsic noise. Bretó *et al.* [3] have noted that environmental noise in the transmission rate can cause such irregularities. Although one could certainly attempt to adapt the distance estimation procedure to such dynamics, our goal was simply to describe a basic procedure and identify some of its limitations.

Estimating the rate of change in the distance

Having a method to estimate the distance to the threshold opens up the possibility of estimating whether the distance to the threshold is changing over time. A simple way to do this is to look at the change in distance estimates between two windows of a time series. Figure S6 shows some results using this simple approach. As one might expect from the variance of the distance estimates apparent in figure 3, the estimated changes in distance were also highly variable (figure S6, bottom). However, the distribution of estimates is dense in the neighborhood of the true value, and estimates with the wrong sign are relatively infrequent. Thus it seems one may be able to be much more confident about statements of whether or not the threshold is getting closer than about estimates of how fast the distance is changing.

References

[1] He D, Ionides EL, King AA, 2010 Plug-and-play inference for disease dynamics: measles in large and small populations as a case study. *J. Roy. Soc. Interface* **7**, 271–283. doi:10.1098/rsif.2009.0151

[2] Martinez-Bakker M, King AA, Rohani P, 2015 Unraveling the transmission ecology of polio. *PLoS Biol.* **13**, e1002172. doi:10.1371/journal.pbio.1002172

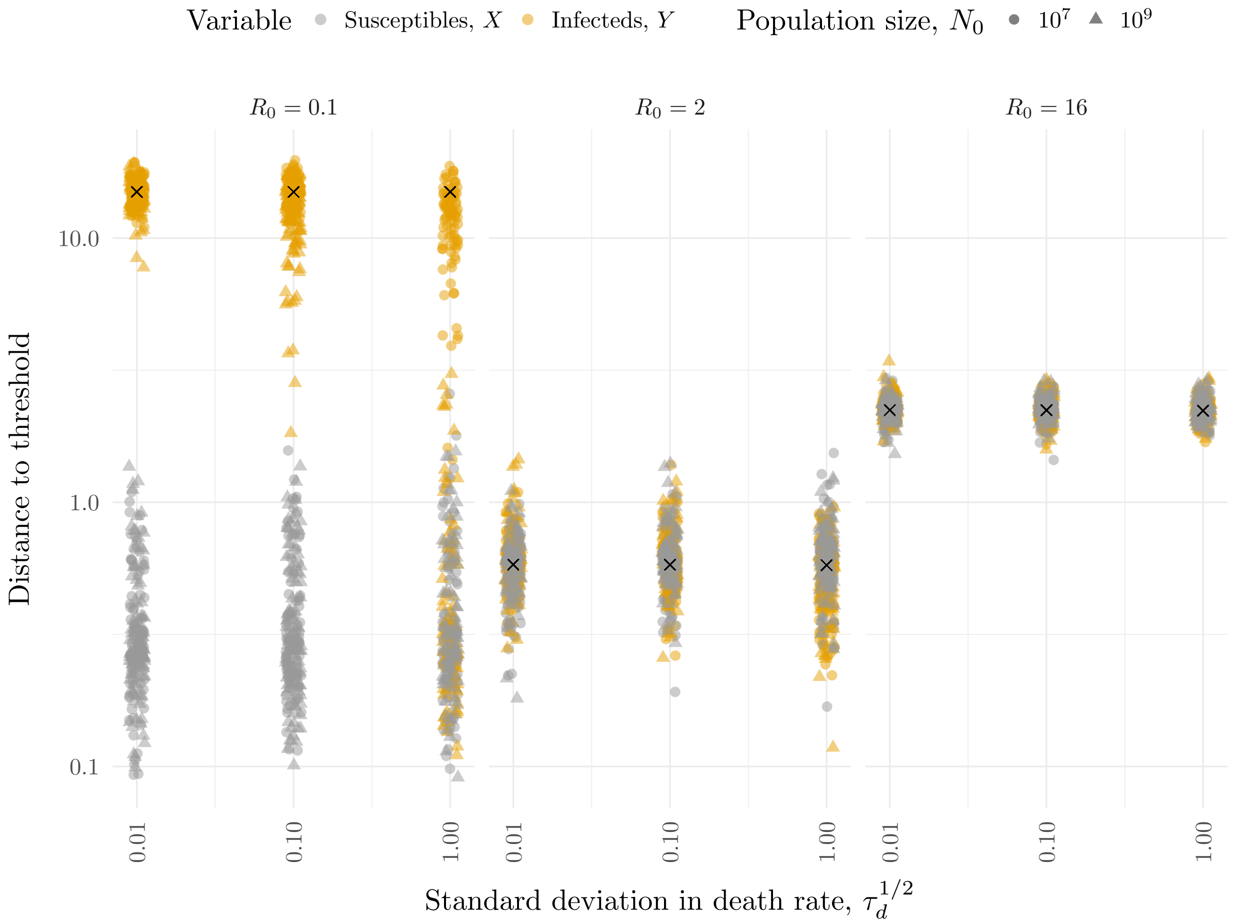

Figure S2: The accuracy of distance estimates is not affected by environmental noise in the death rate parameter until the variation becomes extreme, which is apparent in the case that $R_0 = 0.1$ and $N_0 = 10^9$. The true distance is marked with an '$\times$'. Parameter η was fixed to $1/(3\sqrt{10^7})$. Other parameters were in table 1. Parameter β was set to $R_0(\gamma + \mu)$. The color of the plotted points indicates the state variable on which the estimates were based.

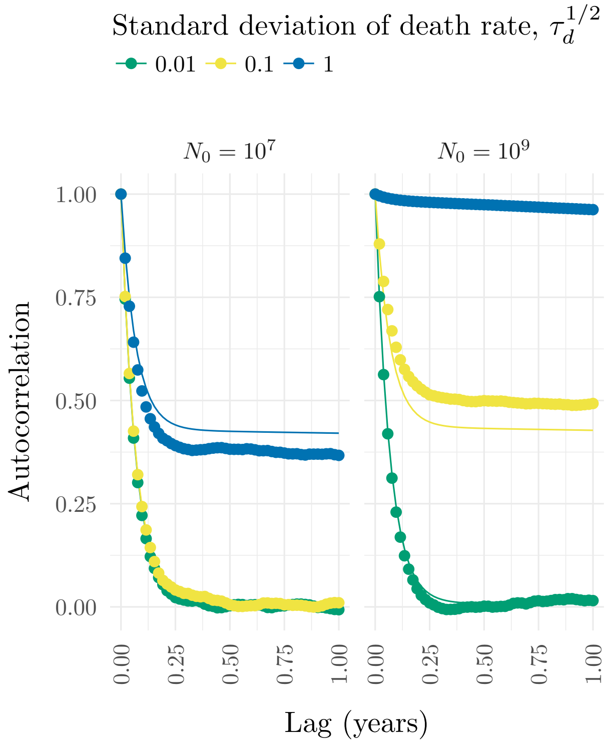

Figure S3: The sensitivity of the autocorrelation of the number infected to environmental noise in the death rate depends on the lag. Points are estimate of the autocorrelation from a long (1000 year) time series simulated according to our Markov process model. Lines represent the analytic calculations based on equation (2.7). Parameter η was fixed to $1/(3\sqrt{10^7})$. Other parameters were as in table 1, with β set to $0.1(\gamma + \mu)$.

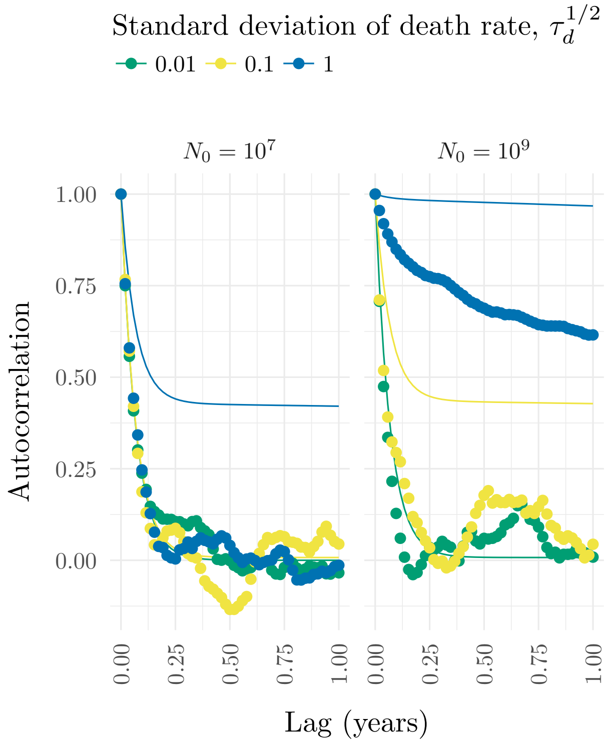

Figure S4: The increase in the autocorrelation with noise in the death rate can be less for 20 year observation periods than the long term average. Points are estimates of the autocorrelation from a 20 year time series simulated according to our Markov process model. Lines represent the analytic calculations based on equation (2.7). The points are much lower than the lines than is the case in figure S3, where the simulations were much longer. Parameter η was fixed to $1/(3\sqrt{10^7})$. Other parameters are in table 1, with β set to $0.1(\gamma+\mu)$.

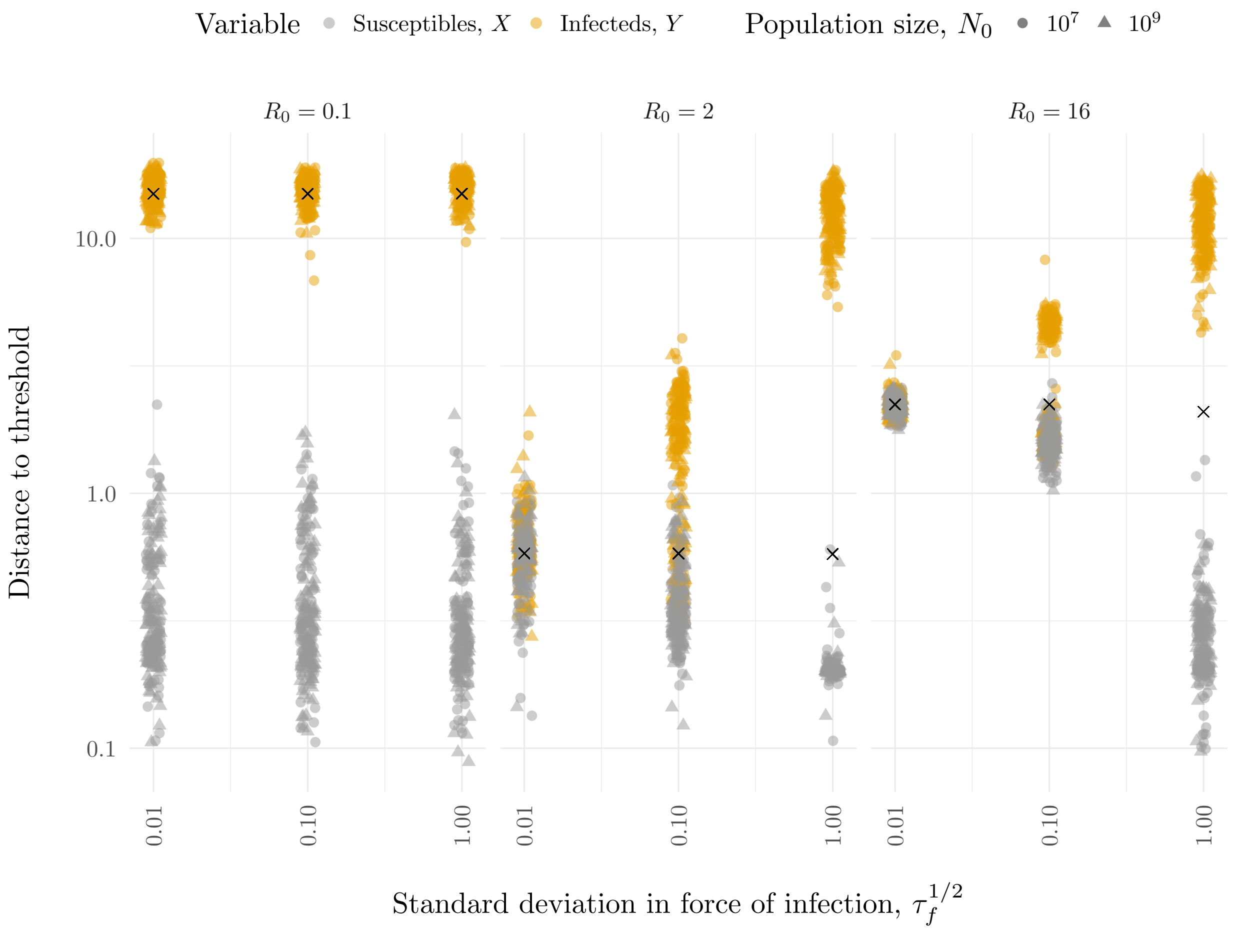

Figure S5: The accuracy of distance estimates is affected by environmental noise in the force of infection when it is large (here, a standard deviation above 0.01) and there are cycles that it can interrupt (here, R_0 of 2 or 16). The true distance is marked with an 'x'. Parameter η was fixed to $1/(3\sqrt{10^7})$. Other parameters are in table 1. β was set to $R_0(\gamma + \mu)$. The color of the plotted points indicates the state variable on which the estimates were based.

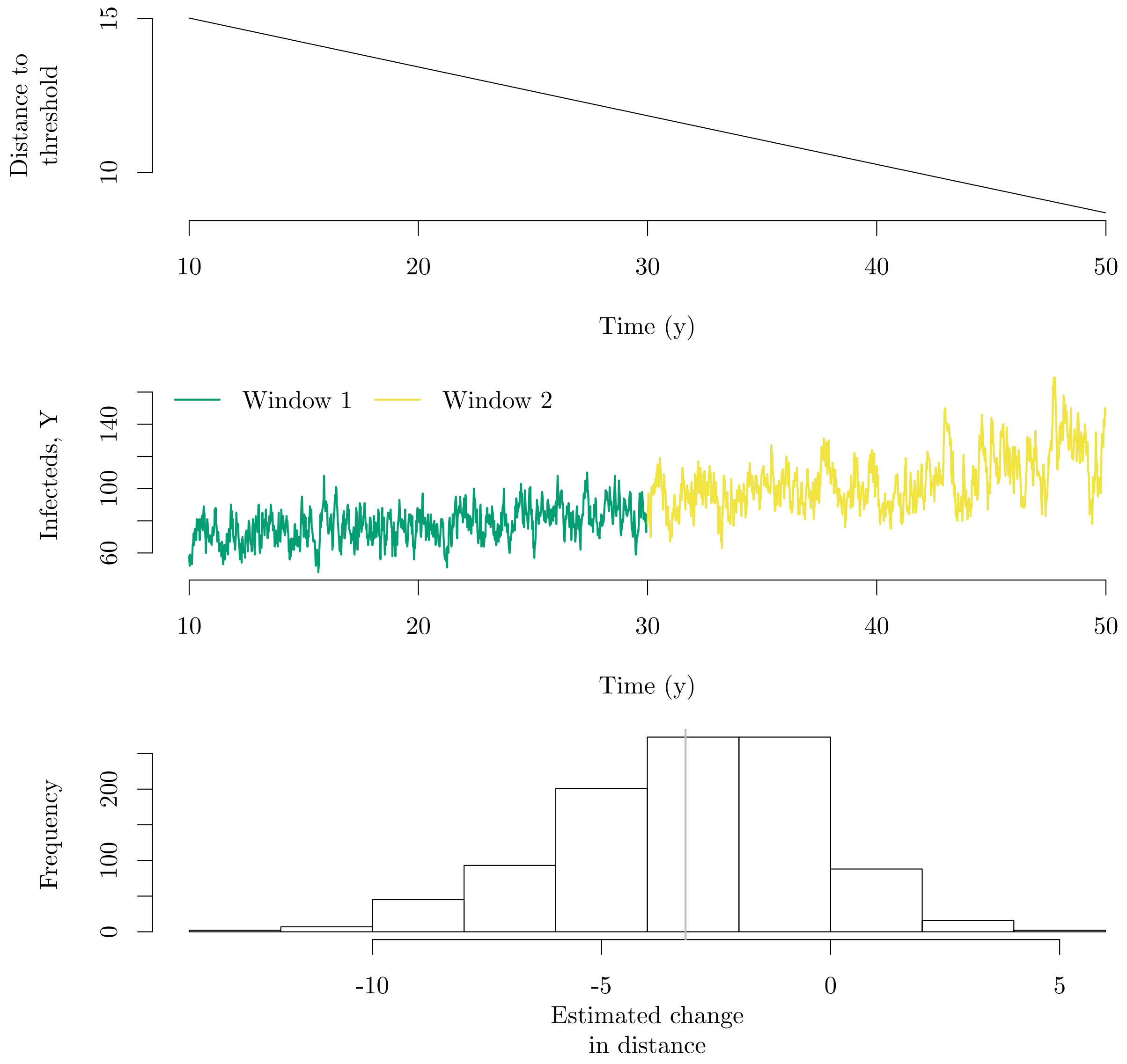

Figure S6: A series of distance estimates can be used to estimate the change in the distance to the threshold as it is being approached. (Top) The true distance to the threshold in the model changes over time due to changes in the transmission rate β. The true distance at a given time is calculated as the informative eigenvalue of the Jacobian evaluated at the equilibrium based on the transmission rate at that time. (Middle) Example of data used to estimate the change in distance. A distance estimate was obtained using the data in each window. (Bottom) The distribution of the estimates of change in distance from 1000 replicates. The gray line marks the change in the true distance between the middle of the windows. We consider this line to mark the true value for the estimated change in distance. Simulation parameters are as in table 1.

[3] Bretó C, He D, Ionides EL, King AA, 2009 Time series analysis via mechanistic models. *Ann. Appl. Stat.* **3**, 319–348. doi:10.1214/08-AOAS201
